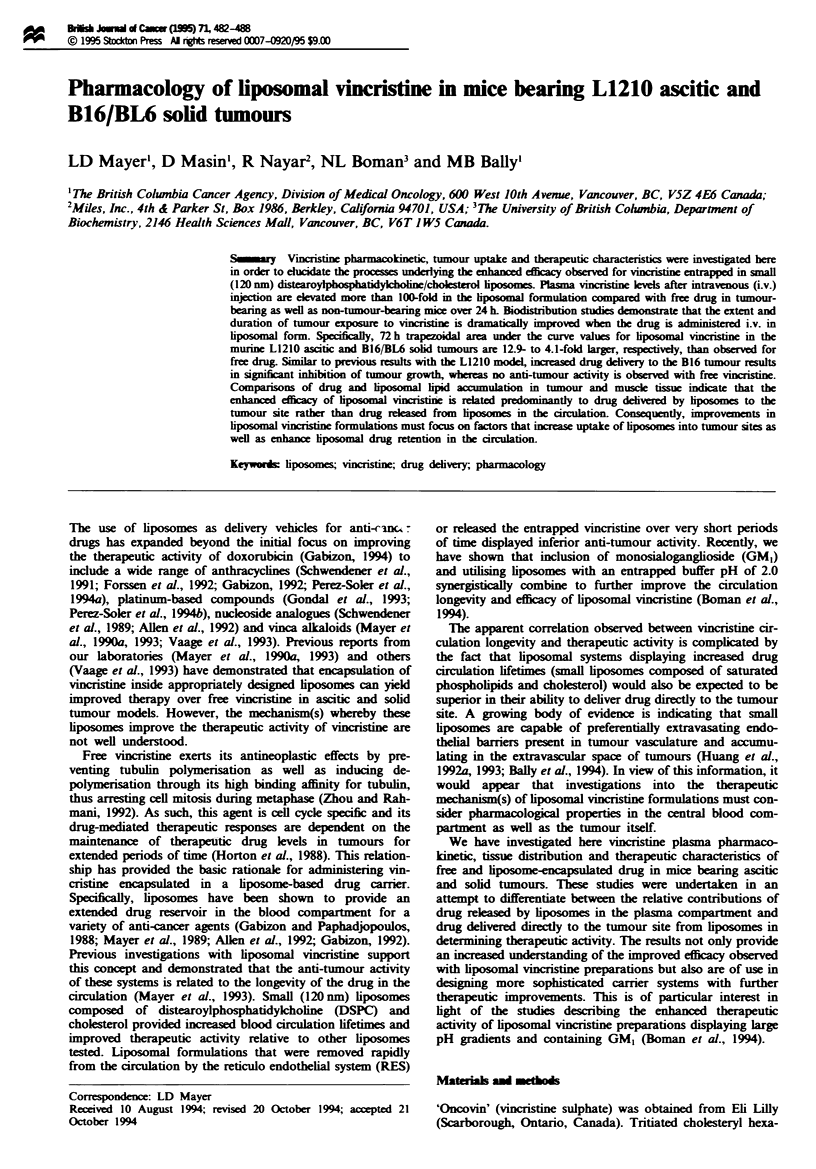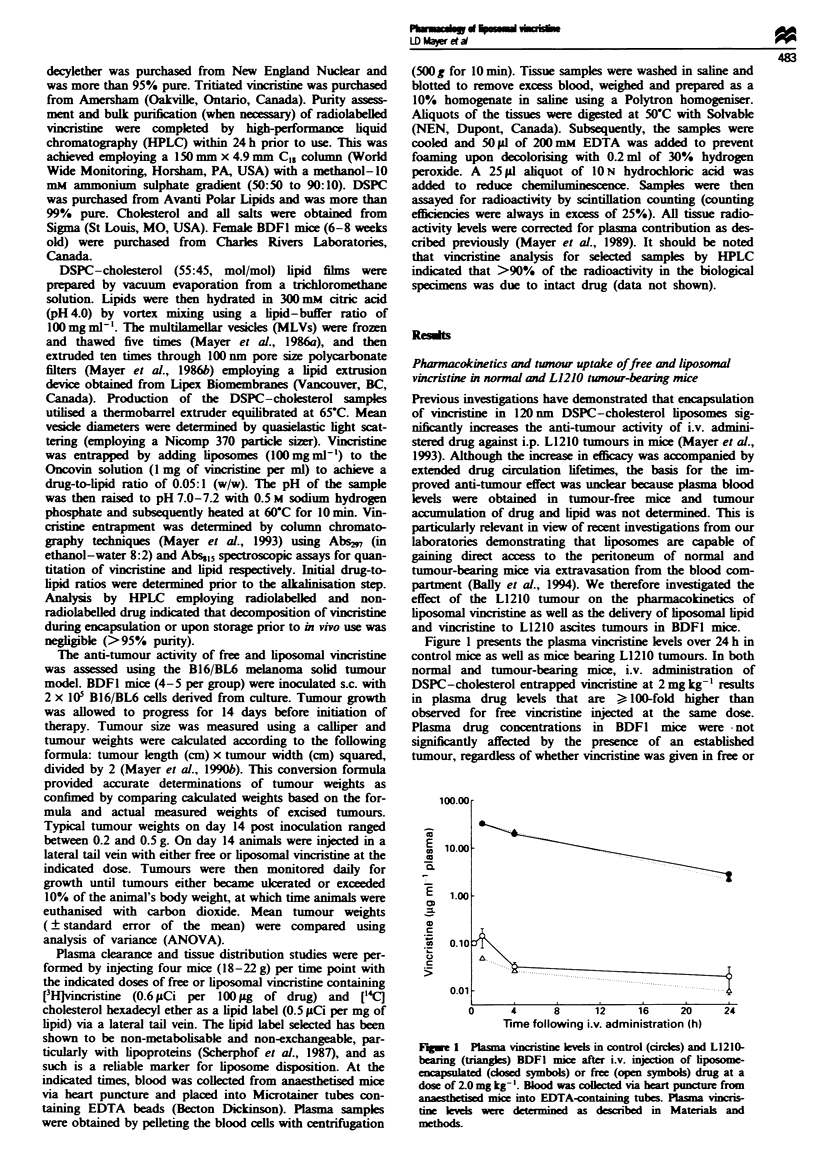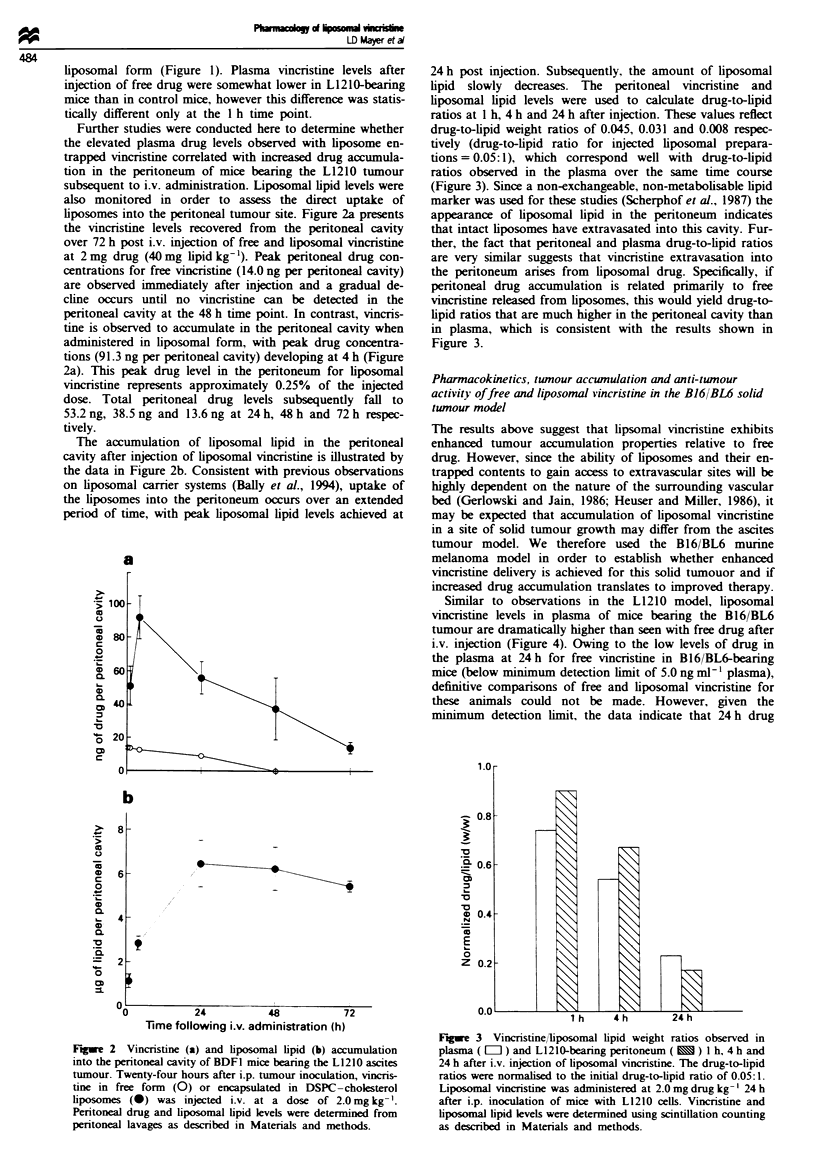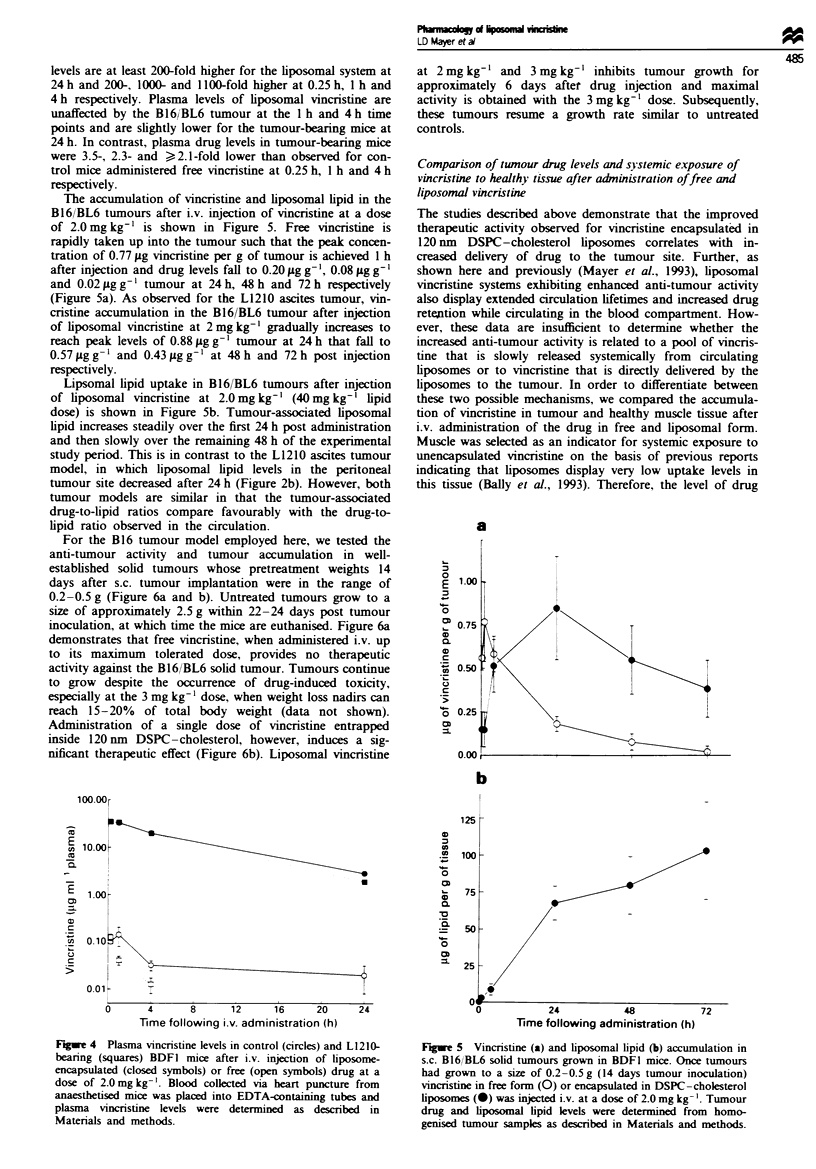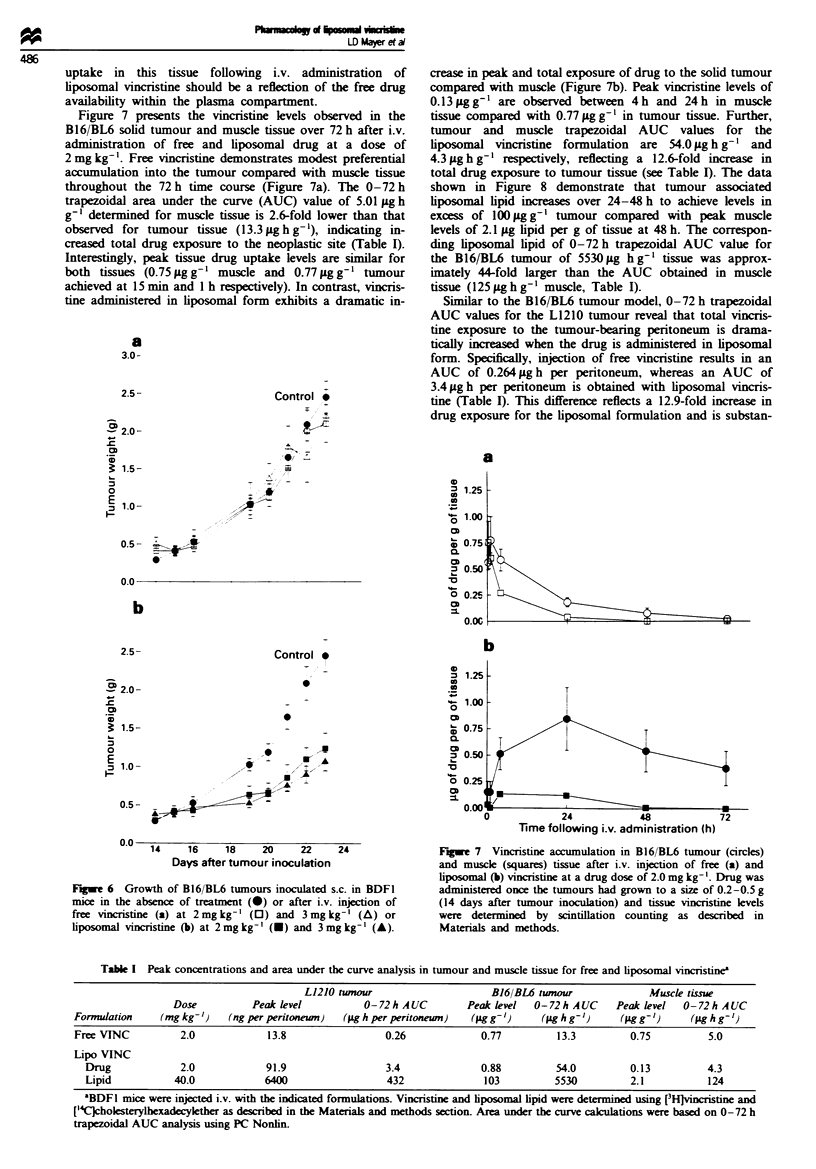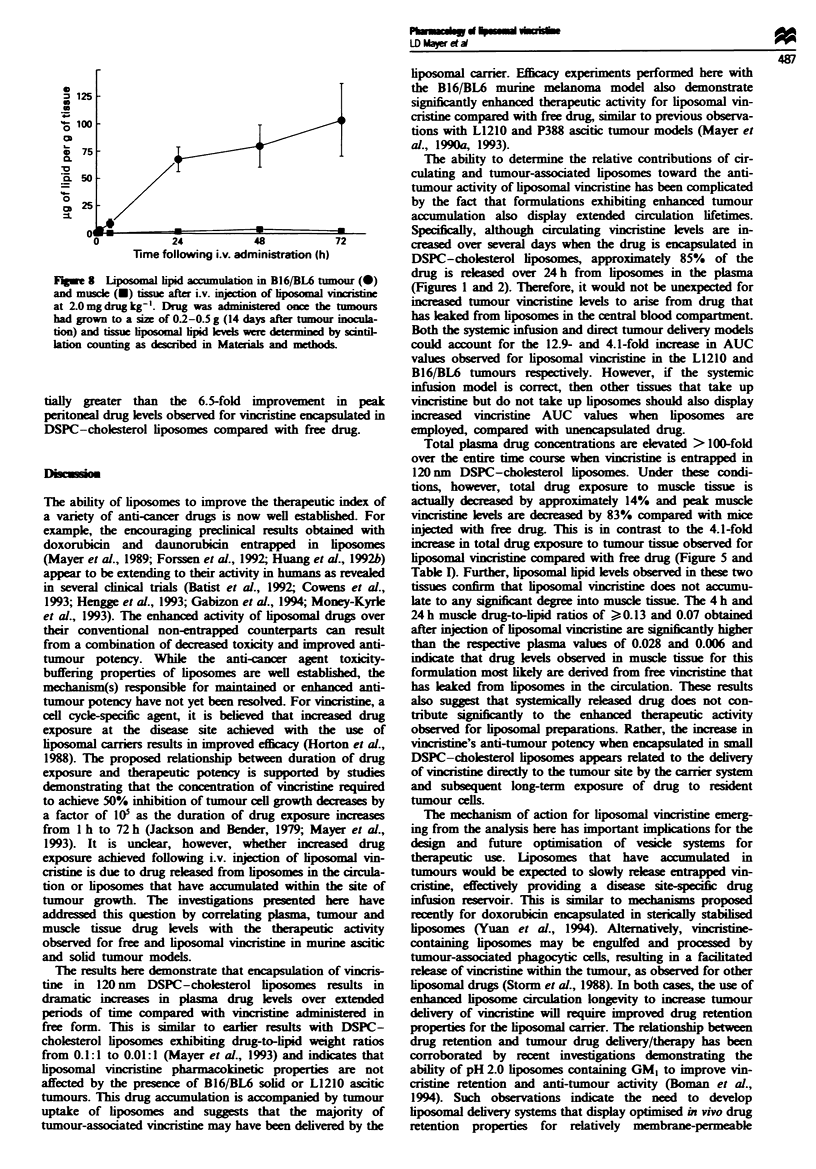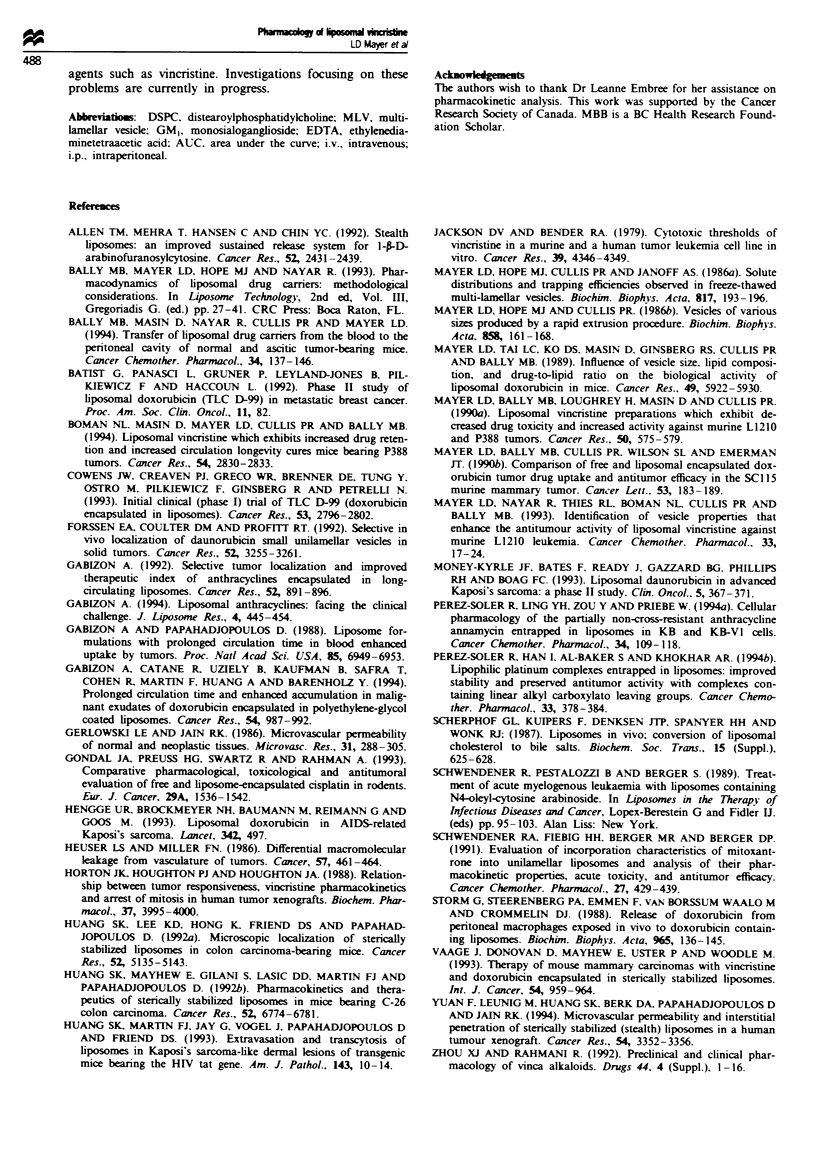# Pharmacology of liposomal vincristine in mice bearing L1210 ascitic and B16/BL6 solid tumours.

**DOI:** 10.1038/bjc.1995.98

**Published:** 1995-03

**Authors:** L. D. Mayer, D. Masin, R. Nayar, N. L. Boman, M. B. Bally

**Affiliations:** British Columbia Cancer Agency, Division of Medical Oncology, Vancouver, Canada.

## Abstract

Vincristine pharmacokinetic, tumour uptake and therapeutic characteristics were investigated here in order to elucidate the processes underlying the enhanced efficacy observed for vincristine entrapped in small (120 nm) distearoylphosphatidylcholine/cholesterol liposomes. Plasma vincristine levels after intravenous (i.v.) injection are elevated more than 100-fold in the liposomal formulation compared with free drug in tumour-bearing as well as non-tumour-bearing mice over 24 h. Biodistribution studies demonstrate that the extent and duration of tumour exposure to vincristine is dramatically improved when the drug is administered i.v. in liposomal form. Specifically, 72 h trapezoidal area under the curve values for liposomal vincristine in the murine L1210 ascitic and B16/BL6 solid tumours are 12.9- to 4.1-fold larger, respectively, than observed for free drug. Similar to previous results with the L1210 model, increased drug delivery to the B16 tumour results in significant inhibition of tumour growth, whereas no anti-tumour activity is observed with free vincristine. Comparisons of drug and liposomal lipid accumulation in tumour and muscle tissue indicate that the enhanced efficacy of liposomal vincristine is related predominantly to drug delivered by liposomes to the tumour site rather than drug released from liposomes in the circulation. Consequently, improvements in liposomal vincristine formulations must focus on factors that increase uptake of liposomes into tumour sites as well as enhance liposomal drug retention in the circulation.


© 1995 Stockton Press All rights reserved 0007-0920/95 $9.00

### Pharmacology of liposomal vincristine in mice bearing L1210 ascitic and B16/BL6 solid tumours

LD Mayer[1], D Masin[1], R Nayar[2], NL Boman[3] and MB Bally[1]

[1]*The British Columbia Cancer Agency, Division of Medical Oncology, 600 West 10th Avenue, Vancouver, BC, V5Z 4E6 Canada;* [2]*Miles, Inc., 4th & Parker St, Box 1986, Berkley, California 94701, USA;* [3]*The University of British Columbia, Department of Biochemistry, 2146 Health Sciences Mall, Vancouver, BC, V6T 1W5 Canada.*

**Summary** Vincristine pharmacokinetic, tumour uptake and therapeutic characteristics were investigated here in order to elucidate the processes underlying the enhanced efficacy observed for vincristine entrapped in small (120 nm) distearoylphosphatidylcholine/cholesterol liposomes. Plasma vincristine levels after intravenous (i.v.) injection are elevated more than 100-fold in the liposomal formulation compared with free drug in tumour-bearing as well as non-tumour-bearing mice over 24 h. Biodistribution studies demonstrate that the extent and duration of tumour exposure to vincristine is dramatically improved when the drug is administered i.v. in liposomal form. Specifically, 72 h trapezoidal area under the curve values for liposomal vincristine in the murine L1210 ascitic and B16/BL6 solid tumours are 12.9- to 4.1-fold larger, respectively, than observed for free drug. Similar to previous results with the L1210 model, increased drug delivery to the B16 tumour results in significant inhibition of tumour growth, whereas no anti-tumour activity is observed with free vincristine. Comparisons of drug and liposomal lipid accumulation in tumour and muscle tissue indicate that the enhanced efficacy of liposomal vincristine is related predominantly to drug delivered by liposomes to the tumour site rather than drug released from liposomes in the circulation. Consequently, improvements in liposomal vincristine formulations must focus on factors that increase uptake of liposomes into tumour sites as well as enhance liposomal drug retention in the circulation.

**Keywords:** liposomes; vincristine; drug delivery; pharmacology

The use of liposomes as delivery vehicles for anti-cancer drugs has expanded beyond the initial focus on improving the therapeutic activity of doxorubicin (Gabizon, 1994) to include a wide range of anthracyclines (Schwendener *et al.*, 1991; Forssen *et al.*, 1992; Gabizon, 1992; Perez-Soler *et al.*, 1994*a*), platinum-based compounds (Gondal *et al.*, 1993; Perez-Soler *et al.*, 1994*b*), nucleoside analogues (Schwendener *et al.*, 1989; Allen *et al.*, 1992) and vinca alkaloids (Mayer *et al.*, 1990*a*, 1993; Vaage *et al.*, 1993). Previous reports from our laboratories (Mayer *et al.*, 1990*a*, 1993) and others (Vaage *et al.*, 1993) have demonstrated that encapsulation of vincristine inside appropriately designed liposomes can yield improved therapy over free vincristine in ascitic and solid tumour models. However, the mechanism(s) whereby these liposomes improve the therapeutic activity of vincristine are not well understood.

Free vincristine exerts its antineoplastic effects by preventing tubulin polymerisation as well as inducing depolymerisation through its high binding affinity for tubulin, thus arresting cell mitosis during metaphase (Zhou and Rahmani, 1992). As such, this agent is cell cycle specific and its drug-mediated therapeutic responses are dependent on the maintenance of therapeutic drug levels in tumours for extended periods of time (Horton *et al.*, 1988). This relationship has provided the basic rationale for administering vincristine encapsulated in a liposome-based drug carrier. Specifically, liposomes have been shown to provide an extended drug reservoir in the blood compartment for a variety of anti-cancer agents (Gabizon and Paphadjopoulos, 1988; Mayer *et al.*, 1989; Allen *et al.*, 1992; Gabizon, 1992). Previous investigations with liposomal vincristine support this concept and demonstrated that the anti-tumour activity of these systems is related to the longevity of the drug in the circulation (Mayer *et al.*, 1993). Small (120 nm) liposomes composed of distearoylphosphatidylcholine (DSPC) and cholesterol provided increased blood circulation lifetimes and improved therapeutic activity relative to other liposomes tested. Liposomal formulations that were removed rapidly from the circulation by the reticulo endothelial system (RES) or released the entrapped vincristine over very short periods of time displayed inferior anti-tumour activity. Recently, we have shown that inclusion of monosialoganglioside ($GM_1$) and utilising liposomes with an entrapped buffer pH of 2.0 synergistically combine to further improve the circulation longevity and efficacy of liposomal vincristine (Boman *et al.*, 1994).

The apparent correlation observed between vincristine circulation longevity and therapeutic activity is complicated by the fact that liposomal systems displaying increased drug circulation lifetimes (small liposomes composed of saturated phospholipids and cholesterol) would also be expected to be superior in their ability to deliver drug directly to the tumour site. A growing body of evidence is indicating that small liposomes are capable of preferentially extravasating endothelial barriers present in tumour vasculature and accumulating in the extravascular space of tumours (Huang *et al.*, 1992*a*, 1993; Bally *et al.*, 1994). In view of this information, it would appear that investigations into the therapeutic mechanism(s) of liposomal vincristine formulations must consider pharmacological properties in the central blood compartment as well as the tumour itself.

We have investigated here vincristine plasma pharmacokinetic, tissue distribution and therapeutic characteristics of free and liposome-encapsulated drug in mice bearing ascitic and solid tumours. These studies were undertaken in an attempt to differentiate between the relative contributions of drug released by liposomes in the plasma compartment and drug delivered directly to the tumour site from liposomes in determining therapeutic activity. The results not only provide an increased understanding of the improved efficacy observed with liposomal vincristine preparations but also are of use in designing more sophisticated carrier systems with further therapeutic improvements. This is of particular interest in light of the studies describing the enhanced therapeutic activity of liposomal vincristine preparations displaying large pH gradients and containing $GM_1$ (Boman *et al.*, 1994).

### Materials and methods

'Oncovin' (vincristine sulphate) was obtained from Eli Lilly (Scarborough, Ontario, Canada). Tritiated cholesteryl hexa-

Correspondence: LD Mayer
Received 10 August 1994; revised 20 October 1994; accepted 21 October 1994

decylether was purchased from New England Nuclear and was more than 95% pure. Tritiated vincristine was purchased from Amersham (Oakville, Ontario, Canada). Purity assessment and bulk purification (when necessary) of radiolabelled vincristine were completed by high-performance liquid chromatography (HPLC) within 24 h prior to use. This was achieved employing a 150 mm × 4.9 mm $C_{18}$ column (World Wide Monitoring, Horsham, PA, USA) with a methanol–10 mM ammonium sulphate gradient (50:50 to 90:10). DSPC was purchased from Avanti Polar Lipids and was more than 99% pure. Cholesterol and all salts were obtained from Sigma (St Louis, MO, USA). Female BDF1 mice (6–8 weeks old) were purchased from Charles Rivers Laboratories, Canada.

DSPC–cholesterol (55:45, mol/mol) lipid films were prepared by vacuum evaporation from a trichloromethane solution. Lipids were then hydrated in 300 mM citric acid (pH 4.0) by vortex mixing using a lipid–buffer ratio of 100 mg $ml^{-1}$. The multilamellar vesicles (MLVs) were frozen and thawed five times (Mayer *et al.*, 1986*a*), and then extruded ten times through 100 nm pore size polycarbonate filters (Mayer *et al.*, 1986*b*) employing a lipid extrusion device obtained from Lipex Biomembranes (Vancouver, BC, Canada). Production of the DSPC–cholesterol samples utilised a thermobarrel extruder equilibrated at 65°C. Mean vesicle diameters were determined by quasielastic light scattering (employing a Nicomp 370 particle sizer). Vincristine was entrapped by adding liposomes (100 mg $ml^{-1}$) to the Oncovin solution (1 mg of vincristine per ml) to achieve a drug-to-lipid ratio of 0.05:1 (w/w). The pH of the sample was then raised to pH 7.0–7.2 with 0.5 M sodium hydrogen phosphate and subsequently heated at 60°C for 10 min. Vincristine entrapment was determined by column chromatography techniques (Mayer *et al.*, 1993) using $Abs_{297}$ (in ethanol–water 8:2) and $Abs_{815}$ spectroscopic assays for quantitation of vincristine and lipid respectively. Initial drug-to-lipid ratios were determined prior to the alkalinisation step. Analysis by HPLC employing radiolabelled and non-radiolabelled drug indicated that decomposition of vincristine during encapsulation or upon storage prior to *in vivo* use was negligible (>95% purity).

The anti-tumour activity of free and liposomal vincristine was assessed using the B16/BL6 melanoma solid tumour model. BDF1 mice (4–5 per group) were inoculated s.c. with $2 \times 10^5$ B16/BL6 cells derived from culture. Tumour growth was allowed to progress for 14 days before initiation of therapy. Tumour size was measured using a calliper and tumour weights were calculated according to the following formula: tumour length (cm) × tumour width (cm) squared, divided by 2 (Mayer *et al.*, 1990*b*). This conversion formula provided accurate determinations of tumour weights as confirmed by comparing calculated weights based on the formula and actual measured weights of excised tumours. Typical tumour weights on day 14 post inoculation ranged between 0.2 and 0.5 g. On day 14 animals were injected in a lateral tail vein with either free or liposomal vincristine at the indicated dose. Tumours were then monitored daily for growth until tumours either became ulcerated or exceeded 10% of the animal's body weight, at which time animals were euthanised with carbon dioxide. Mean tumour weights (± standard error of the mean) were compared using analysis of variance (ANOVA).

Plasma clearance and tissue distribution studies were performed by injecting four mice (18–22 g) per time point with the indicated doses of free or liposomal vincristine containing [$^3$H]vincristine (0.6 μCi per 100 μg of drug) and [$^{14}$C] cholesterol hexadecyl ether as a lipid label (0.5 μCi per mg of lipid) via a lateral tail vein. The lipid label selected has been shown to be non-metabolisable and non-exchangeable, particularly with lipoproteins (Scherphof *et al.*, 1987), and as such is a reliable marker for liposome disposition. At the indicated times, blood was collected from anaesthetised mice via heart puncture and placed into Microtainer tubes containing EDTA beads (Becton Dickinson). Plasma samples were obtained by pelleting the blood cells with centrifugation (500 *g* for 10 min). Tissue samples were washed in saline and blotted to remove excess blood, weighed and prepared as a 10% homogenate in saline using a Polytron homogeniser. Aliquots of the tissues were digested at 50°C with Solvable (NEN, Dupont, Canada). Subsequently, the samples were cooled and 50 μl of 200 mM EDTA was added to prevent foaming upon decolorising with 0.2 ml of 30% hydrogen peroxide. A 25 μl aliquot of 10 N hydrochloric acid was added to reduce chemiluminescence. Samples were then assayed for radioactivity by scintillation counting (counting efficiencies were always in excess of 25%). All tissue radioactivity levels were corrected for plasma contribution as described previously (Mayer *et al.*, 1989). It should be noted that vincristine analysis for selected samples by HPLC indicated that >90% of the radioactivity in the biological specimens was due to intact drug (data not shown).

### Results

### *Pharmacokinetics and tumour uptake of free and liposomal vincristine in normal and L1210 tumour-bearing mice*

Previous investigations have demonstrated that encapsulation of vincristine in 120 nm DSPC–cholesterol liposomes significantly increases the anti-tumour activity of i.v. administered drug against i.p. L1210 tumours in mice (Mayer *et al.*, 1993). Although the increase in efficacy was accompanied by extended drug circulation lifetimes, the basis for the improved anti-tumour effect was unclear because plasma blood levels were obtained in tumour-free mice and tumour accumulation of drug and lipid was not determined. This is particularly relevant in view of recent investigations from our laboratories demonstrating that liposomes are capable of gaining direct access to the peritoneum of normal and tumour-bearing mice via extravasation from the blood compartment (Bally *et al.*, 1994). We therefore investigated the effect of the L1210 tumour on the pharmacokinetics of liposomal vincristine as well as the delivery of liposomal lipid and vincristine to L1210 ascites tumours in BDF1 mice.

Figure 1 presents the plasma vincristine levels over 24 h in control mice as well as mice bearing L1210 tumours. In both normal and tumour-bearing mice, i.v. administration of DSPC–cholesterol entrapped vincristine at 2 mg $kg^{-1}$ results in plasma drug levels that are ⩾100-fold higher than observed for free vincristine injected at the same dose. Plasma drug concentrations in BDF1 mice were not significantly affected by the presence of an established tumour, regardless of whether vincristine was given in free or

**Figure 1** Plasma vincristine levels in control (circles) and L1210-bearing (triangles) BDF1 mice after i.v. injection of liposome-encapsulated (closed symbols) or free (open symbols) drug at a dose of 2.0 mg $kg^{-1}$. Blood was collected via heart puncture from anaesthetised mice into EDTA-containing tubes. Plasma vincristine levels were determined as described in Materials and methods.

liposomal form (Figure 1). Plasma vincristine levels after injection of free drug were somewhat lower in L1210-bearing mice than in control mice, however this difference was statistically different only at the 1 h time point.

Further studies were conducted here to determine whether the elevated plasma drug levels observed with liposome entrapped vincristine correlated with increased drug accumulation in the peritoneum of mice bearing the L1210 tumour subsequent to i.v. administration. Liposomal lipid levels were also monitored in order to assess the direct uptake of liposomes into the peritoneal tumour site. Figure 2a presents the vincristine levels recovered from the peritoneal cavity over 72 h post i.v. injection of free and liposomal vincristine at 2 mg drug (40 mg lipid $kg^{-1}$). Peak peritoneal drug concentrations for free vincristine (14.0 ng per peritoneal cavity) are observed immediately after injection and a gradual decline occurs until no vincristine can be detected in the peritoneal cavity at the 48 h time point. In contrast, vincristine is observed to accumulate in the peritoneal cavity when administered in liposomal form, with peak drug concentrations (91.3 ng per peritoneal cavity) developing at 4 h (Figure 2a). This peak drug level in the peritoneum for liposomal vincristine represents approximately 0.25% of the injected dose. Total peritoneal drug levels subsequently fall to 53.2 ng, 38.5 ng and 13.6 ng at 24 h, 48 h and 72 h respectively.

The accumulation of liposomal lipid in the peritoneal cavity after injection of liposomal vincristine is illustrated by the data in Figure 2b. Consistent with previous observations on liposomal carrier systems (Bally *et al.*, 1994), uptake of the liposomes into the peritoneum occurs over an extended period of time, with peak liposomal lipid levels achieved at 24 h post injection. Subsequently, the amount of liposomal lipid slowly decreases. The peritoneal vincristine and liposomal lipid levels were used to calculate drug-to-lipid ratios at 1 h, 4 h and 24 h after injection. These values reflect drug-to-lipid weight ratios of 0.045, 0.031 and 0.008 respectively (drug-to-lipid ratio for injected liposomal preparations = 0.05:1), which correspond well with drug-to-lipid ratios observed in the plasma over the same time course (Figure 3). Since a non-exchangeable, non-metabolisable lipid marker was used for these studies (Scherphof *et al.*, 1987) the appearance of liposomal lipid in the peritoneum indicates that intact liposomes have extravasated into this cavity. Further, the fact that peritoneal and plasma drug-to-lipid ratios are very similar suggests that vincristine extravasation into the peritoneum arises from liposomal drug. Specifically, if peritoneal drug accumulation is related primarily to free vincristine released from liposomes, this would yield drug-to-lipid ratios that are much higher in the peritoneal cavity than in plasma, which is consistent with the results shown in Figure 3.

**Figure 2** Vincristine (**a**) and liposomal lipid (**b**) accumulation into the peritoneal cavity of BDF1 mice bearing the L1210 ascites tumour. Twenty-four hours after i.p. tumour inoculation, vincristine in free form (○) or encapsulated in DSPC–cholesterol liposomes (●) was injected i.v. at a dose of 2.0 mg $kg^{-1}$. Peritoneal drug and liposomal lipid levels were determined from peritoneal lavages as described in Materials and methods.

### *Pharmacokinetics, tumour accumulation and anti-tumour activity of free and liposomal vincristine in the B16/BL6 solid tumour model*

The results above suggest that lipsomal vincristine exhibits enhanced tumour accumulation properties relative to free drug. However, since the ability of liposomes and their entrapped contents to gain access to extravascular sites will be highly dependent on the nature of the surrounding vascular bed (Gerlowski and Jain, 1986; Heuser and Miller, 1986), it may be expected that accumulation of liposomal vincristine in a site of solid tumour growth may differ from the ascites tumour model. We therefore used the B16/BL6 murine melanoma model in order to establish whether enhanced vincristine delivery is achieved for this solid tumouor and if increased drug accumulation translates to improved therapy.

Similar to observations in the L1210 model, liposomal vincristine levels in plasma of mice bearing the B16/BL6 tumour are dramatically higher than seen with free drug after i.v. injection (Figure 4). Owing to the low levels of drug in the plasma at 24 h for free vincristine in B16/BL6-bearing mice (below minimum detection limit of 5.0 ng $ml^{-1}$ plasma), definitive comparisons of free and liposomal vincristine for these animals could not be made. However, given the minimum detection limit, the data indicate that 24 h drug

**Figure 3** Vincristine/liposomal lipid weight ratios observed in plasma ( □ ) and L1210-bearing peritoneum ( ▨ ) 1 h, 4 h and 24 h after i.v. injection of liposomal vincristine. The drug-to-lipid ratios were normalised to the initial drug-to-lipid ratio of 0.05:1. Liposomal vincristine was administered at 2.0 mg drug $kg^{-1}$ 24 h after i.p. inoculation of mice with L1210 cells. Vincristine and liposomal lipid levels were determined using scintillation counting as described in Materials and methods.

levels are at least 200-fold higher for the liposomal system at 24 h and 200-, 1000- and 1100-fold higher at 0.25 h, 1 h and 4 h respectively. Plasma levels of liposomal vincristine are unaffected by the B16/BL6 tumour at the 1 h and 4 h time points and are slightly lower for the tumour-bearing mice at 24 h. In contrast, plasma drug levels in tumour-bearing mice were 3.5-, 2.3- and $\geqslant$2.1-fold lower than observed for control mice administered free vincristine at 0.25 h, 1 h and 4 h respectively.

The accumulation of vincristine and liposomal lipid in the B16/BL6 tumours after i.v. injection of vincristine at a dose of 2.0 mg $kg^{-1}$ is shown in Figure 5. Free vincristine is rapidly taken up into the tumour such that the peak concentration of 0.77 μg vincristine per g of tumour is achieved 1 h after injection and drug levels fall to 0.20 μg $g^{-1}$, 0.08 μg $g^{-1}$ and 0.02 μg $g^{-1}$ tumour at 24 h, 48 h and 72 h respectively (Figure 5a). As observed for the L1210 ascites tumour, vincristine accumulation in the B16/BL6 tumour after injection of liposomal vincristine at 2 mg $kg^{-1}$ gradually increases to reach peak levels of 0.88 μg $g^{-1}$ tumour at 24 h that fall to 0.57 μg $g^{-1}$ and 0.43 μg $g^{-1}$ at 48 h and 72 h post injection respectively.

Lipsomal lipid uptake in B16/BL6 tumours after injection of liposomal vincristine at 2.0 mg $kg^{-1}$ (40 mg $kg^{-1}$ lipid dose) is shown in Figure 5b. Tumour-associated liposomal lipid increases steadily over the first 24 h post administration and then slowly over the remaining 48 h of the experimental study period. This is in contrast to the L1210 ascites tumour model, in which liposomal lipid levels in the peritoneal tumour site decreased after 24 h (Figure 2b). However, both tumour models are similar in that the tumour-associated drug-to-lipid ratios compare favourably with the drug-to-lipid ratio observed in the circulation.

For the B16 tumour model employed here, we tested the anti-tumour activity and tumour accumulation in well-established solid tumours whose pretreatment weights 14 days after s.c. tumour implantation were in the range of 0.2–0.5 g (Figure 6a and b). Untreated tumours grow to a size of approximately 2.5 g within 22–24 days post tumour inoculation, at which time the mice are euthanised. Figure 6a demonstrates that free vincristine, when administered i.v. up to its maximum tolerated dose, provides no therapeutic activity against the B16/BL6 solid tumour. Tumours continue to grow despite the occurrence of drug-induced toxicity, especially at the 3 mg $kg^{-1}$ dose, when weight loss nadirs can reach 15–20% of total body weight (data not shown). Administration of a single dose of vincristine entrapped inside 120 nm DSPC-cholesterol, however, induces a significant therapeutic effect (Figure 6b). Liposomal vincristine at 2 mg $kg^{-1}$ and 3 mg $kg^{-1}$ inhibits tumour growth for approximately 6 days after drug injection and maximal activity is obtained with the 3 mg $kg^{-1}$ dose. Subsequently, these tumours resume a growth rate similar to untreated controls.

*Comparison of tumour drug levels and systemic exposure of vincristine to healthy tissue after administration of free and liposomal vincristine*

The studies described above demonstrate that the improved therapeutic activity observed for vincristine encapsulated in 120 nm DSPC-cholesterol liposomes correlates with increased delivery of drug to the tumour site. Further, as shown here and previously (Mayer *et al.*, 1993), liposomal vincristine systems exhibiting enhanced anti-tumour activity also display extended circulation lifetimes and increased drug retention while circulating in the blood compartment. However, these data are insufficient to determine whether the increased anti-tumour activity is related to a pool of vincristine that is slowly released systemically from circulating liposomes or to vincristine that is directly delivered by the liposomes to the tumour. In order to differentiate between these two possible mechanisms, we compared the accumulation of vincristine in tumour and healthy muscle tissue after i.v. administration of the drug in free and liposomal form. Muscle was selected as an indicator for systemic exposure to unencapsulated vincristine on the basis of previous reports indicating that liposomes display very low uptake levels in this tissue (Bally *et al.*, 1993). Therefore, the level of drug

**Figure 4** Plasma vincristine levels in control (circles) and L1210-bearing (squares) BDF1 mice after i.v. injection of liposome-encapsulated (closed symbols) or free (open symbols) drug at a dose of 2.0 mg $kg^{-1}$. Blood collected via heart puncture from anaesthetised mice was placed into EDTA-containing tubes and plasma vincristine levels were determined as described in Materials and methods.

**Figure 5** Vincristine (a) and liposomal lipid (b) accumulation in s.c. B16/BL6 solid tumours grown in BDF1 mice. Once tumours had grown to a size of 0.2–0.5 g (14 days tumour inoculation) vincristine in free form (○) or encapsulated in DSPC-cholesterol liposomes (●) was injected i.v. at a dose of 2.0 mg $kg^{-1}$. Tumour drug and liposomal lipid levels were determined from homogenised tumour samples as described in Materials and methods.

uptake in this tissue following i.v. administration of liposomal vincristine should be a reflection of the free drug availability within the plasma compartment.

Figure 7 presents the vincristine levels observed in the B16/BL6 solid tumour and muscle tissue over 72 h after i.v. administration of free and liposomal drug at a dose of 2 mg $kg^{-1}$. Free vincristine demonstrates modest preferential accumulation into the tumour compared with muscle tissue throughout the 72 h time course (Figure 7a). The 0–72 h trapezoidal area under the curve (AUC) value of 5.01 μg h $g^{-1}$ determined for muscle tissue is 2.6-fold lower than that observed for tumour tissue (13.3 μg h $g^{-1}$), indicating increased total drug exposure to the neoplastic site (Table I). Interestingly, peak tissue drug uptake levels are similar for both tissues (0.75 μg $g^{-1}$ muscle and 0.77 μg $g^{-1}$ tumour achieved at 15 min and 1 h respectively). In contrast, vincristine administered in liposomal form exhibits a dramatic increase in peak and total exposure of drug to the solid tumour compared with muscle (Figure 7b). Peak vincristine levels of 0.13 μg $g^{-1}$ are observed between 4 h and 24 h in muscle tissue compared with 0.77 μg $g^{-1}$ in tumour tissue. Further, tumour and muscle trapezoidal AUC values for the liposomal vincristine formulation are 54.0 μg h $g^{-1}$ and 4.3 μg h $g^{-1}$ respectively, reflecting a 12.6-fold increase in total drug exposure to tumour tissue (see Table I). The data shown in Figure 8 demonstrate that tumour associated liposomal lipid increases over 24–48 h to achieve levels in excess of 100 μg $g^{-1}$ tumour compared with peak muscle levels of 2.1 μg lipid per g of tissue at 48 h. The corresponding liposomal lipid of 0–72 h trapezoidal AUC value for the B16/BL6 tumour of 5530 μg h $g^{-1}$ tissue was approximately 44-fold larger than the AUC obtained in muscle tissue (125 μg h $g^{-1}$ muscle, Table I).

Similar to the B16/BL6 tumour model, 0–72 h trapezoidal AUC values for the L1210 tumour reveal that total vincristine exposure to the tumour-bearing peritoneum is dramatically increased when the drug is administered in liposomal form. Specifically, injection of free vincristine results in an AUC of 0.264 μg h per peritoneum, whereas an AUC of 3.4 μg h per peritoneum is obtained with liposomal vincristine (Table I). This difference reflects a 12.9-fold increase in drug exposure for the liposomal formulation and is substan-

**Figure 6** Growth of B16/BL6 tumours inoculated s.c. in BDF1 mice in the absence of treatment (●) or after i.v. injection of free vincristine (**a**) at 2 mg $kg^{-1}$ (□) and 3 mg $kg^{-1}$ (Δ) or liposomal vincristine (**b**) at 2 mg $kg^{-1}$ (■) and 3 mg $kg^{-1}$ (▲).

**Figure 7** Vincristine accumulation in B16/BL6 tumour (circles) and muscle (squares) tissue after i.v. injection of free (**a**) and liposomal (**b**) vincristine at a drug dose of 2.0 mg $kg^{-1}$. Drug was administered once the tumours had grown to a size of 0.2–0.5 g (14 days after tumour inoculation) and tissue vincristine levels were determined by scintillation counting as described in Materials and methods.

**Table I** Peak concentrations and area under the curve analysis in tumour and muscle tissue for free and liposomal vincristine[a]

| | | *L1210 tumour* | | *B16/BL6 tumour* | | *Muscle tissue* | |
|---|---|---|---|---|---|---|---|
| *Formulation* | *Dose (mg $kg^{-1}$)* | *Peak level (ng per peritoneum)* | *0–72 h AUC (μg h per peritoneum)* | *Peak level (μg $g^{-1}$)* | *0–72 h AUC (μg h $g^{-1}$)* | *Peak level (μg $g^{-1}$)* | *0–72 h AUC (μg h $g^{-1}$)* |
| Free VINC | 2.0 | 13.8 | 0.26 | 0.77 | 13.3 | 0.75 | 5.0 |
| Lipo VINC | | | | | | | |
| Drug | 2.0 | 91.9 | 3.4 | 0.88 | 54.0 | 0.13 | 4.3 |
| Lipid | 40.0 | 6400 | 432 | 103 | 5530 | 2.1 | 124 |

[a]BDF1 mice were injected i.v. with the indicated formulations. Vincristine and liposomal lipid were determined using [$^3$H]vincristine and [$^{14}$C]cholesterylhexadecylether as described in the Materials and methods section. Area under the curve calculations were based on 0–72 h trapezoidal AUC analysis using PC Nonlin.

**Figure 8** Liposomal lipid accumulation in B16/BL6 tumour (●) and muscle (■) tissue after i.v. injection of liposomal vincristine at 2.0 mg drug $kg^{-1}$. Drug was administered once the tumours had grown to a size of 0.2–0.5 g (14 days after tumour inoculation) and tissue liposomal lipid levels were determined by scintillation counting as described in Materials and methods.

tially greater than the 6.5-fold improvement in peak peritoneal drug levels observed for vincristine encapsulated in DSPC–cholesterol liposomes compared with free drug.

### Discussion

The ability of liposomes to improve the therapeutic index of a variety of anti-cancer drugs is now well established. For example, the encouraging preclinical results obtained with doxorubicin and daunorubicin entrapped in liposomes (Mayer *et al.*, 1989; Forssen *et al.*, 1992; Huang *et al.*, 1992*b*) appear to be extending to their activity in humans as revealed in several clinical trials (Batist *et al.*, 1992; Cowens *et al.*, 1993; Hengge *et al.*, 1993; Gabizon *et al.*, 1994; Money-Kyrle *et al.*, 1993). The enhanced activity of liposomal drugs over their conventional non-entrapped counterparts can result from a combination of decreased toxicity and improved anti-tumour potency. While the anti-cancer agent toxicity-buffering properties of liposomes are well established, the mechanism(s) responsible for maintained or enhanced anti-tumour potency have not yet been resolved. For vincristine, a cell cycle-specific agent, it is believed that increased drug exposure at the disease site achieved with the use of liposomal carriers results in improved efficacy (Horton *et al.*, 1988). The proposed relationship between duration of drug exposure and therapeutic potency is supported by studies demonstrating that the concentration of vincristine required to achieve 50% inhibition of tumour cell growth decreases by a factor of $10^5$ as the duration of drug exposure increases from 1 h to 72 h (Jackson and Bender, 1979; Mayer *et al.*, 1993). It is unclear, however, whether increased drug exposure achieved following i.v. injection of liposomal vincristine is due to drug released from liposomes in the circulation or liposomes that have accumulated within the site of tumour growth. The investigations presented here have addressed this question by correlating plasma, tumour and muscle tissue drug levels with the therapeutic activity observed for free and liposomal vincristine in murine ascitic and solid tumour models.

The results here demonstrate that encapsulation of vincristine in 120 nm DSPC–cholesterol liposomes results in dramatic increases in plasma drug levels over extended periods of time compared with vincristine administered in free form. This is similar to earlier results with DSPC–cholesterol liposomes exhibiting drug-to-lipid weight ratios from 0.1:1 to 0.01:1 (Mayer *et al.*, 1993) and indicates that liposomal vincristine pharmacokinetic properties are not affected by the presence of B16/BL6 solid or L1210 ascitic tumours. This drug accumulation is accompanied by tumour uptake of liposomes and suggests that the majority of tumour-associated vincristine may have been delivered by the liposomal carrier. Efficacy experiments performed here with the B16/BL6 murine melanoma model also demonstrate significantly enhanced therapeutic activity for liposomal vincristine compared with free drug, similar to previous observations with L1210 and P388 ascitic tumour models (Mayer *et al.*, 1990*a*, 1993).

The ability to determine the relative contributions of circulating and tumour-associated liposomes toward the anti-tumour activity of liposomal vincristine has been complicated by the fact that formulations exhibiting enhanced tumour accumulation also display extended circulation lifetimes. Specifically, although circulating vincristine levels are increased over several days when the drug is encapsulated in DSPC–cholesterol liposomes, approximately 85% of the drug is released over 24 h from liposomes in the plasma (Figures 1 and 2). Therefore, it would not be unexpected for increased tumour vincristine levels to arise from drug that has leaked from liposomes in the central blood compartment. Both the systemic infusion and direct tumour delivery models could account for the 12.9- and 4.1-fold increase in AUC values observed for liposomal vincristine in the L1210 and B16/BL6 tumours respectively. However, if the systemic infusion model is correct, then other tissues that take up vincristine but do not take up liposomes should also display increased vincristine AUC values when liposomes are employed, compared with unencapsulated drug.

Total plasma drug concentrations are elevated >100-fold over the entire time course when vincristine is entrapped in 120 nm DSPC–cholesterol liposomes. Under these conditions, however, total drug exposure to muscle tissue is actually decreased by approximately 14% and peak muscle vincristine levels are decreased by 83% compared with mice injected with free drug. This is in contrast to the 4.1-fold increase in total drug exposure to tumour tissue observed for liposomal vincristine compared with free drug (Figure 5 and Table I). Further, liposomal lipid levels observed in these two tissues confirm that liposomal vincristine does not accumulate to any significant degree into muscle tissue. The 4 h and 24 h muscle drug-to-lipid ratios of ≥0.13 and 0.07 obtained after injection of liposomal vincristine are significantly higher than the respective plasma values of 0.028 and 0.006 and indicate that drug levels observed in muscle tissue for this formulation most likely are derived from free vincristine that has leaked from liposomes in the circulation. These results also suggest that systemically released drug does not contribute significantly to the enhanced therapeutic activity observed for liposomal preparations. Rather, the increase in vincristine's anti-tumour potency when encapsulated in small DSPC–cholesterol liposomes appears related to the delivery of vincristine directly to the tumour site by the carrier system and subsequent long-term exposure of drug to resident tumour cells.

The mechanism of action for liposomal vincristine emerging from the analysis here has important implications for the design and future optimisation of vesicle systems for therapeutic use. Liposomes that have accumulated in tumours would be expected to slowly release entrapped vincristine, effectively providing a disease site-specific drug infusion reservoir. This is similar to mechanisms proposed recently for doxorubicin encapsulated in sterically stabilised liposomes (Yuan *et al.*, 1994). Alternatively, vincristine-containing liposomes may be engulfed and processed by tumour-associated phagocytic cells, resulting in a facilitated release of vincristine within the tumour, as observed for other liposomal drugs (Storm *et al.*, 1988). In both cases, the use of enhanced liposome circulation longevity to increase tumour delivery of vincristine will require improved drug retention properties for the liposomal carrier. The relationship between drug retention and tumour drug delivery/therapy has been corroborated by recent investigations demonstrating the ability of pH 2.0 liposomes containing $GM_1$ to improve vincristine retention and anti-tumour activity (Boman *et al.*, 1994). Such observations indicate the need to develop liposomal delivery systems that display optimised *in vivo* drug retention properties for relatively membrane-permeable agents such as vincristine. Investigations focusing on these problems are currently in progress.

**Abbreviations**: DSPC, distearoylphosphatidylcholine; MLV, multilamellar vesicle; $GM_1$, monosialoganglioside; EDTA, ethylenediaminetetraacetic acid; AUC, area under the curve; i.v., intravenous; i.p., intraperitoneal.

**Acknowledgements**
The authors wish to thank Dr Leanne Embree for her assistance on pharmacokinetic analysis. This work was supported by the Cancer Research Society of Canada. MBB is a BC Health Research Foundation Scholar.

**References**

ALLEN TM, MEHRA T, HANSEN C AND CHIN YC. (1992). Stealth liposomes: an improved sustained release system for 1-β-D-arabinofuranosylcytosine. *Cancer Res.*, **52**, 2431–2439.

BALLY MB, MAYER LD, HOPE MJ AND NAYAR R. (1993). Pharmacodynamics of liposomal drug carriers: methodological considerations. In *Liposome Technology*, 2nd ed, Vol. III, Gregoriadis G. (ed.) pp. 27–41. CRC Press: Boca Raton, FL.

BALLY MB, MASIN D, NAYAR R, CULLIS PR AND MAYER LD. (1994). Transfer of liposomal drug carriers from the blood to the peritoneal cavity of normal and ascitic tumor-bearing mice. *Cancer Chemother. Pharmacol.*, **34**, 137–146.

BATIST G, PANASCI L, GRUNER P, LEYLAND-JONES B, PILKIEWICZ F AND HACCOUN L. (1992). Phase II study of liposomal doxorubicin (TLC D-99) in metastatic breast cancer. *Proc. Am. Soc. Clin. Oncol.*, **11**, 82.

BOMAN NL, MASIN D, MAYER LD, CULLIS PR AND BALLY MB. (1994). Liposomal vincristine which exhibits increased drug retention and increased circulation longevity cures mice bearing P388 tumors. *Cancer Res.*, **54**, 2830–2833.

COWENS JW, CREAVEN PJ, GRECO WR, BRENNER DE, TUNG Y, OSTRO M, PILKIEWICZ F, GINSBERG R AND PETRELLI N. (1993). Initial clinical (phase I) trial of TLC D-99 (doxorubicin encapsulated in liposomes). *Cancer Res.*, **53**, 2796–2802.

FORSSEN EA, COULTER DM AND PROFFITT RT. (1992). Selective in vivo localization of daunorubicin small unilamellar vesicles in solid tumors. *Cancer Res.*, **52**, 3255–3261.

GABIZON A. (1992). Selective tumor localization and improved therapeutic index of anthracyclines encapsulated in long-circulating liposomes. *Cancer Res.*, **52**, 891–896.

GABIZON A. (1994). Liposomal anthracyclines: facing the clinical challenge. *J. Liposome Res.*, **4**, 445–454.

GABIZON A AND PAPAHADJOPOULOS D. (1988). Liposome formulations with prolonged circulation time in blood enhanced uptake by tumors. *Proc. Natl Acad Sci. USA*, **85**, 6949–6953.

GABIZON A, CATANE R, UZIELY B, KAUFMAN B, SAFRA T, COHEN R, MARTIN F, HUANG A AND BARENHOLZ Y. (1994). Prolonged circulation time and enhanced accumulation in malignant exudates of doxorubicin encapsulated in polyethylene-glycol coated liposomes. *Cancer Res.*, **54**, 987–992.

GERLOWSKI LE AND JAIN RK. (1986). Microvascular permeability of normal and neoplastic tissues. *Microvasc. Res.*, **31**, 288–305.

GONDAL JA, PREUSS HG, SWARTZ R AND RAHMAN A. (1993). Comparative pharmacological, toxicological and antitumoral evaluation of free and liposome-encapsulated cisplatin in rodents. *Eur. J. Cancer*, **29A**, 1536–1542.

HENGGE UR, BROCKMEYER NH, BAUMANN M, REIMANN G AND GOOS M. (1993). Liposomal doxorubicin in AIDS-related Kaposi's sarcoma. *Lancet*, **342**, 497.

HEUSER LS AND MILLER FN. (1986). Differential macromolecular leakage from vasculature of tumors. *Cancer*, **57**, 461–464.

HORTON JK, HOUGHTON PJ AND HOUGHTON JA. (1988). Relationship between tumor responsiveness, vincristine pharmacokinetics and arrest of mitosis in human tumor xenografts. *Biochem. Pharmacol.*, **37**, 3995–4000.

HUANG SK, LEE KD, HONG K, FRIEND DS AND PAPAHADJOPOULOS D. (1992*a*). Microscopic localization of sterically stabilized liposomes in colon carcinoma-bearing mice. *Cancer Res.*, **52**, 5135–5143.

HUANG SK, MAYHEW E, GILANI S, LASIC DD, MARTIN FJ AND PAPAHADJOPOULOS D. (1992*b*). Pharmacokinetics and therapeutics of sterically stabilized liposomes in mice bearing C-26 colon carcinoma. *Cancer Res.*, **52**, 6774–6781.

HUANG SK, MARTIN FJ, JAY G, VOGEL J, PAPAHADJOPOULOS D AND FRIEND DS. (1993). Extravasation and transcytosis of liposomes in Kaposi's sarcoma-like dermal lesions of transgenic mice bearing the HIV tat gene. *Am. J. Pathol.*, **143**, 10–14.

JACKSON DV AND BENDER RA. (1979). Cytotoxic thresholds of vincristine in a murine and a human tumor leukemia cell line in vitro. *Cancer Res.*, **39**, 4346–4349.

MAYER LD, HOPE MJ, CULLIS PR AND JANOFF AS. (1986*a*). Solute distributions and trapping efficiencies observed in freeze-thawed multi-lamellar vesicles. *Biochim. Biophys. Acta*, **817**, 193–196.

MAYER LD, HOPE MJ AND CULLIS PR. (1986*b*). Vesicles of various sizes produced by a rapid extrusion procedure. *Biochim. Biophys. Acta*, **858**, 161–168.

MAYER LD, TAI LC, KO DS, MASIN D, GINSBERG RS, CULLIS PR AND BALLY MB. (1989). Influence of vesicle size, lipid composition, and drug-to-lipid ratio on the biological activity of liposomal doxorubicin in mice. *Cancer Res.*, **49**, 5922–5930.

MAYER LD, BALLY MB, LOUGHREY H, MASIN D AND CULLIS PR. (1990*a*). Liposomal vincristine preparations which exhibit decreased drug toxicity and increased activity against murine L1210 and P388 tumors. *Cancer Res.*, **50**, 575–579.

MAYER LD, BALLY MB, CULLIS PR, WILSON SL AND EMERMAN JT. (1990*b*). Comparison of free and liposomal encapsulated doxorubicin tumor drug uptake and antitumor efficacy in the SC115 murine mammary tumor. *Cancer Lett.*, **53**, 183–189.

MAYER LD, NAYAR R, THIES RL, BOMAN NL, CULLIS PR AND BALLY MB. (1993). Identification of vesicle properties that enhance the antitumour activity of liposomal vincristine against murine L1210 leukemia. *Cancer Chemother. Pharmacol.*, **33**, 17–24.

MONEY-KYRLE JF, BATES F, READY J, GAZZARD BG, PHILLIPS RH AND BOAG FC. (1993). Liposomal daunorubicin in advanced Kaposi's sarcoma: a phase II study. *Clin. Oncol.*, **5**, 367–371.

PEREZ-SOLER R, LING YH, ZOU Y AND PRIEBE W. (1994*a*). Cellular pharmacology of the partially non-cross-resistant anthracycline annamycin entrapped in liposomes in KB and KB-V1 cells. *Cancer Chemother. Pharmacol.*, **34**, 109–118.

PEREZ-SOLER R, HAN I, AL-BAKER S AND KHOKHAR AR. (1994*b*). Lipophilic platinum complexes entrapped in liposomes: improved stability and preserved antitumor activity with complexes containing linear alkyl carboxylato leaving groups. *Cancer Chemother. Pharmacol.*, **33**, 378–384.

SCHERPHOF GL, KUIPERS F, DENKSEN JTP, SPANYER HH AND WONK RJ; (1987). Liposomes in vivo; conversion of liposomal cholesterol to bile salts. *Biochem. Soc. Trans.*, **15** (Suppl.), 625–628.

SCHWENDENER R, PESTALOZZI B AND BERGER S. (1989). Treatment of acute myelogenous leukaemia with liposomes containing N4-oleyl-cytosine arabinoside. In *Liposomes in the Therapy of Infectious Diseases and Cancer*, Lopex-Berestein G and Fidler IJ. (eds) pp. 95–103. Alan Liss: New York.

SCHWENDENER RA, FIEBIG HH, BERGER MR AND BERGER DP. (1991). Evaluation of incorporation characteristics of mitoxantrone into unilamellar liposomes and analysis of their pharmacokinetic properties, acute toxicity, and antitumor efficacy. *Cancer Chemother. Pharmacol.*, **27**, 429–439.

STORM G, STEERENBERG PA, EMMEN F, VAN BORSSUM WAALO M AND CROMMELIN DJ. (1988). Release of doxorubicin from peritoneal macrophages exposed in vivo to doxorubicin containing liposomes. *Biochim. Biophys. Acta*, **965**, 136–145.

VAAGE J, DONOVAN D, MAYHEW E, USTER P AND WOODLE M. (1993). Therapy of mouse mammary carcinomas with vincristine and doxorubicin encapsulated in sterically stabilized liposomes. *Int. J. Cancer*, **54**, 959–964.

YUAN F, LEUNIG M, HUANG SK, BERK DA, PAPAHADJOPOULOS D AND JAIN RK. (1994). Microvascular permeability and interstitial penetration of sterically stabilized (stealth) liposomes in a human tumour xenograft. *Cancer Res.*, **54**, 3352–3356.

ZHOU XJ AND RAHMANI R. (1992). Preclinical and clinical pharmacology of vinca alkaloids. *Drugs 44*, **4** (Suppl.), 1–16.